# Some Phases of Albuminuria

**Published:** 1903-01

**Authors:** Willim S. Gordon

**Affiliations:** Richmond, Va., Professor of Practice of Medicine, University College of Medicine


### SOME PHASES OF ALBUMINURIA.*

By WILLIM S. GORDON, M.D., Richmond, Va.,
Professor of Practice of Medicine, University College of Medicine.

Neither the subject of this paper nor the conclusions arrived at are new, but the following cases may throw additional light upon certain mooted points in connection with the appearance of albumin in the urine:

Case I.—A healthy-looking young man of good physique and excellent habits was examined for life insurance and informed that his urine contained a trace of albumin. My examination confirmed the discovery and revealed a few small hyaline casts. The patient's mother suffers from gout, and he himself has had slight manifestations of uricacidemia. He had been exercising vigorously at boat-rowing. A quiet life and regulation of his diet resulted in a prompt disappearance of the morbid urinary conditions.

Case II.—A young man apparently in good health applied to me for life insurance examination. The urine contained a trace of albumin. He was a free meat-eater. Restriction of the diet to vegetables, fruits and easily digested articles caused a disappear-

---

*Read before the Richmond Academy of Medicine and Surgery, November 25, 1902.

ance of the albumin, an increase of flesh and strength, and a marked improvement in physical and mental vigor.

CASE III.—A young man was refused insurance on the grounds above mentioned and placed himself under my care. He was troubled with intestinal indigestion. Regulation of his diet, meats being interdicted, was followed by marked improvement in his health and a normal condition of the urine. In this case there was, in addition to a small amount of albumin, an abundance of octahedral calcium oxalate crystals. His father, who indulges rather freely in flesh food, has albumin and casts in his urine at present.

CASE IV.—A young man of nervous temperament and with a tendency to hypochondriasis was found to have intermittent albuminuria. After repeated examinations of the urine I discovered uric acid and small hyaline casts. His general appearance was indicative of perfect health. He had been quite an athlete. The measures adopted in Case I. and the assurance that he did not have chronic or acute nephritis resulted in the establishment of normal urine.

CASE V.—A young man, a physician, consulted me for slight albuminuria, which he stated was intermittent and ordinarily present only after assuming the upright position. I failed to find albumin or casts on several examinations. His life required a good deal of physical exertion. The usual treatment produced the usual results.

CASE VI.—A young professional man came under my treatment for albuminuria. Casts were present at intervals. Strict dieting produced little difference in his condition. A faint mitral murmur of insufficiency was finally detected. Rest was followed by almost complete return of the urine to the normal.

CASE VII.—A young primipara who has been under my observation for some weeks and has had considerable renal disturbance was delivered a few days ago of a dead-born child at the beginning of the ninth month. The induction of labor had been contemplated but was postponed on account of the subsidence of mild uremic symptoms. At the time of labor the urine contained one half volume of albumin and a number of casts, mostly hyaline but partly granular and epithelial. The urea amounted to fifteen grains in the ounce, and had been increasing along with the albumin. Nature intervened and settled the question of responsibility.

Several of these cases illustrate what has been variously termed physiological, cyclic, intermittent, periodic, and functional albuminuria. Albumin is at times accidentally discovered in the urine and doubtless has frequently been present and disappeared without producing symptoms that could be recognized either by patient or physician. The condition has come and gone like a thief in the night; but the questions arise, has any loss been entailed or any damage done, and what were the circumstances favorable to the thief's visit? There are careful clinicians and pathologists holding the view that transient and slight albuminuria is physiological, but the more I study these cases the more am I convinced that albumin in the urine, whether it be in small amount or of short duration, is pathological.

Let us take a reasonable, scientific view of the question. If a meal over-rich in proteids be ingested and albumin be found leaking out of the renal blood-vessels in the glomerulus, it would seem that two pathological processes are taking place—first, imperfect digestion and assimilation, with its necessary attendant, faulty metabolism; and second, an impairment of the epithelium in the capillary tuft or the enclosing capsule. Physiology teaches and proves that complete or partial anemia of the tuft is followed by albuminuria; and it appears reasonable to believe that albumin does not occur in urine unless the secreting and excreting renal structures are morbidly affected. If this be true, physiological albuminuria is a misnomer, whether it be the result of functional disturbance or of organic changes. Function is physiological; impairment of function is pathological in the correct acceptance and definition of the term.

In studying the cases cited it will be observed that too much exercise, too much proteid food, nervous disorder, cardiac disease, and bodily position, were responsible, either singly or combined, for the albuminuria. In one instance the patient's safety-valve action of the right heart relieved him of cardiac or pulmonary congestion, but caused a passive renal congestion. In another instance mitral insufficiency, so faint as almost to defy detection, resulted likewise. Another patient was irritating his kidney epithelium with crystals of uric acid and calcium oxalate. In every instance the physiological processes of the body were more or less crippled, and the consequence was a morbid or pathological condition, no matter how slight, how transient, or how harmless for the time it lasted.

If, accordingly, transient albuminuria is not to be regarded as a normal occurrence, then it should be regarded as an indication of disordered health, and should receive the careful treatment of the physician, for there are good grounds for believing that the frequent recurrence or the long continuance of such conditions will eventually lead to permanent and possibly organic disease. In every case of albuminuria, diligent search ought to be made for its various causes, and the examiner for life-insurance, in particular, should not recommend an applicant who has even a trace of albumin in his urine, on the ground that it can be considered a physiological condition.

In a letter received recently from the medical director of a life-insurance company, the writer expresses his belief that a certain number of cases of slight albuminuria are due to unconfessed or unrecognized syphilis. That this disease may produce amyloid degeneration of the kidney is well known, and some authorities claim that specific infection is responsible for certain cases of cyclic, intermittent, or slight permanent albuminuria. On the other hand, we have just seen that many other causes can be assigned. My own belief is that a large proportion of the cases under discussion are due to uricacidemia or to some kind of gastro-intestinal disorder.

The last case reported is interesting from the fact that, contrary to what is usually observed in such patients, the urea and albumin both increased in amount. The problem to solve related not so much to the danger of eclampsia, for uremic symptoms, as stated, had disappeared, but to the probability of persisting renal disease after the uterus was emptied. We are all convinced that albumin and large casts may be present for some length of time in the urine and disappear, leaving a healthy kidney; and that absence of albumin and even of casts is occasionally noted during the course of chronic interstitial nephritis. I can recall a marked illustration of the latter truth, the patient having uremic insanity, yet the urine being, at intervals, free from albumin and casts. We are thus led to believe that albumin and casts may not be of serious significance, but that diminution of urea is always to be regarded as a dangerous condition, although the symptoms may tolerate considerable amounts of this excretion product.

It may not be amiss, in this connection, to express my belief that the experiment of injecting urea into animals in order to prove its comparative innocuousness is unscientific and untrustworthy. Let urea be injected into a human subject whose kidneys are diseased and unable to excrete it, and I venture to assert that a prompt exhibition of its injurious effects will follow.

Another truth it seems to me is taught by this case of pregnancy. It is that albumin is, in a great measure, taken from the blood in the glomerulus of the kidney, and that urea is excreted from the neck of the tubule around which the second set of vessels is entwined. Such is the teaching of physiology, proven by experiment and corroborated by pathology and clinical observation. Albumin and hyaline casts are not desirable and ought to receive immediate attention, but decrease in the amount of urea is the most important sign in the pathology and treatment of renal disease.

